# Editorial: Spring Hippocampal Research Conference and Beyond

**DOI:** 10.3389/fnmol.2021.773308

**Published:** 2021-10-12

**Authors:** James A. Ainge, Mariangela Chisari, Akiva Cohen, Steven J. Mennerick, Lisa Topolnik, Jochen C. Meier

**Affiliations:** ^1^School of Psychology and Neuroscience, University of St Andrews, St Andrews, United Kingdom; ^2^Section of Pharmacology, Department of Biomedical and Biotechnological Sciences, University of Catania, Catania, Italy; ^3^University of Pennsylvania and Children's Hospital of Philadelphia, Philadelphia, PA, United States; ^4^Department of Psychiatry, Taylor Family Institute for Innovative Psychiatric Research, Washington University in St. Louis School of Medicine, St. Louis, MO, United States; ^5^Department of Biochemistry, Microbiology and Bio-Informatics, Laval University, Québec City, QC, Canada; ^6^Neuroscience Axis, CHU de Québec Research Center (CHUL), Québec City, QC, Canada; ^7^Division Cell Physiology, Technical University Braunschweig, Zoological Institute, Braunschweig, Germany

**Keywords:** hippocampus, memory, mood, neurotransmitters, development

The Spring Hippocampal Research Conference is devoted to all aspects of hippocampal structure, connectivity, function, and malfunction. It is an open meeting built around small symposia proposed by the participants. The meeting brings together some of the leading figures in hippocampal research but also provides an opportunity for early career researchers to present to a specialist audience. As such the meeting tackles the latest issues in hippocampal research and provides one of the most important forums in the world for discussing all aspects of the hippocampus. It has been held every other year since 1988, except 2021 due to Covid-19 pandemic. While it does not make up for the meeting we all missed in 2021, this special issue has provided an opportunity for researchers to present recent research in rodents, primates and humans examining the hippocampus from all of these different perspectives.

The special issue contains a number of articles examining the role of the hippocampus in spatial memory and navigation. Bretas et al. examined place and reward signals in the monkey hippocampus using a virtual navigation task. They showed that, as in rodents, place cells in the monkey hippocampus represent overlapping path sections using distinct ensembles which also code for various characteristics of reward. Two imaging studies present findings of how the human hippocampus processes different aspects of spatial memory. Faulmann et al. show that cognitive map retrieval is predominantly associated with posterior parahippocampal rather than hippocampal activation. They went on to show that this activation is significantly greater during direction but not distance estimation. Dahmani et al. examined the relationship between hippocampal volume and performance on both navigation and olfactory identification tasks. They found that right fimbria-fornix volume was correlated with both tasks in participants who used hippocampus-based spatial memory strategies.

The special issue also includes articles examining hippocampal oscillations. Grossberg presents a comprehensive review of spatial representations within the hippocampal-entorhinal network together with a theoretical synthesis that explains many findings regarding theta oscillations within the hippocampus.

Social recognition is fundamental for social decision making and the establishment of long-lasting affiliative behaviors. The study of Cymerblit-Sabba et al. revealed that social recognition and preference rely on activation of the CA2 hippocampal subfield *via* the hypothalamic paraventricular nucleus, which can represent a part of an evolutionary conserved neural circuitry for formation of social memory. Furthermore, encoding of social memory is regulated by oxytocin acting through the oxytocin receptor (Oxtr). Young and Song examined the neuronal expression of Oxtr transcripts in the dorsal hippocampus. The study revealed that in addition to CA2 and CA3 principal cells, Oxtr is present in different types of inhibitory interneurons, pointing to a potentially important role of oxytocin in coordination of hippocampal network activity by modulating inhibitory circuits.

The region-specific differences in the modulation of synaptic transmission along the dorso-ventral axis of the hippocampus remain in focus of research. Recently, Dubovyk and Manahan-Vaughan reported a much higher expression of dopamine receptor 2 in the ventral portion of the hippocampus, which can support the region-specific synaptic plasticity and dopamine-dependent behavioral outcomes. Furthermore, Trompoukis and Papatheodoropoulos revealed a different contribution of the GABA-B receptors to heterosynaptic depression between the two hippocampal regions.

There were and still are many studies highlighting a critical role of the hippocampal formation in neuropsychiatric disorders. In this issue, we paid particular attention to Alzheimer disease (AD), epilepsy, aging and cognitive decline, psychosis and schizophrenia, multiple sclerosis, and traumatic brain injury.

In particular, rewiring and synaptic reorganization in the dentate gyrus after brain injury were discussed by Del Turco et al. The authors studied re-innervation of the denervated dentate gyrus in organotypic tissue cultures of the entorhinal cortex and hippocampus. They found that the majority of sprouting associational calretinin-positive axons are mossy cell axons—nearly the entire dentate gyrus entorhinal target zone was re-innervated by sprouting of associational and commissural mossy cell axons.

Along this line, by deleting BDNF receptor TrkB from serotonergic neurons in the adult brain, Sahu et al. investigated the effects of increased brain serotonin levels on energy metabolism and learning and memory. In spite of increased food intake, the transgenic mice were significantly leaner than their wildtype littermates. The results suggest that loss of the TrkB receptor in the 5-HT neurons increases 5-HT levels, thereby regulating neuronal plasticity and behavior. Furthermore, reduction of TrkB in 5-HT neurons increased proliferation, but not long-term survival, of hippocampal cells that was consistent with increase in immature neuronal markers such as doublecortin and calretinin in the transgenic animals.

Bartsch and Behr used N-methyl-D-aspartate receptor (NMDAR) antagonist MK-801 to model first-episode psychosis in rats and studied long-term potentiation (LTP) in subicular regular-firing cells in acute hippocampal slices. The authors provide evidence for a non-canonical postsynaptic NMDAR-independent LTP in ventral subicular but not in CA1 regular-firing pyramidal cells, which was dependent on D1/D5 dopamine receptor activation, postsynaptic Ca^2+^ signaling and activation of protein kinase A. This aberrant form of LTP in ventral subicular regular-firing neurons was suggested to interfere with physiological hippocampal output processing and to contribute to hippocampal dysfunction in psychotic events.

Manzella et al. examined another negative modulator of NMDA receptor function, ketamine, when administered in early life, simulating pediatric anesthetic use. Previous work has shown that anesthetics produce neurotoxicity in brain areas like the subiculum and adverse neurocognitive outcomes. Here, the authors showed that subiculum-related gamma oscillations during non-REM sleep were altered by early ketamine exposure when assayed in adolescence. Synaptic plasticity, measured as LTP, was also disrupted. However, the authors found no overt disruption of sleep macrostructure. The results offer a potential functional substrate for neurocognitive impairments observed with early exposure to anesthetics.

In contrast to the classical role of neurotransmitter dysregulation in disease Shen et al. provided evidence for a novel role of sarcosine (aka N-methylglycine; a glycine transporter 1 GlyT1 inhibitor) as antiepileptic drug. The authors previously demonstrated in rodent epilepsy models that augmentation of glycine suppressed chronic seizures and altered acute seizure thresholds. In this study, they developed a rapid hippocampal kindling model to produce stable epileptogenesis and demonstrated a resultant overexpression of GlyT1 and dysregulated DNA methylation in kindled rats. They further provided experimental evidence that sarcosine can delay kindling epileptogenesis, which was associated with altered DNA methylation. Sarcosine treatment during kindling changed hippocampal 5 mC and 5 hmC levels and modified the expression levels of the demethylase TET1.

Disinhibition can be important for different forms of hippocampal learning, and its structural or functional alterations can be involved in cognitive decline associated with aging. However, how the properties of cells making up the disinhibitory circuits may change during aging remains unknown. Francavilla et al. discover the age-dependent changes in morphological and physiological properties of vasoactive intestinal peptide (VIP)-expressing, type 3 interneuron-specific (IS3) GABAergic neurons that reside in the CA1 hippocampus. The age-dependent changes in the intrinsic properties and the firing of IS3 cells occur in parallel with changes in the inhibitory drive received by their postsynaptic GABAergic targets. These data provide first evidence on the age-dependent remodeling in intrinsic properties of a disinhibitory cell type in the hippocampus and demonstrate an overall increase in inhibition of inhibitory interneurons, with a possible hyperactivity of CA1 pyramidal cells.

Karunakaran researches behavioral methods for determining if an individual will develop Alzheimer's disease at a later stage. A mouse model for Alzheimer's disease, APPswe/PS1dE9 (APP/PS1) displays amyloidosis and is a model for Alzheimer's disease-like symptoms. Previous studies have attempted to study cognitive deficits in these mice, but these previous experiments lack sensitivity and specificity. This study analyzes the results of the Morris Water Maze behavioral test in different ways that allow for increased specificity in behavioral testing. This study found that even if APP/PS1 mice had a similar success rate on the final day of testing, they had more unsuccessful trials during training to get to that result. This study also examined the strategies that the mice used to find the platform. Some of these strategies were spatially based, meaning that they used the hippocampus. Some of these strategies were non-spatial, meaning that they were used independent of the hippocampus. When tested, the wild-type mice and APP/PS1 employed different sequences of learning strategies to find the platform. Specifically, many of the APP/PS1 mice employed a non-spatial strategy called circling. This study demonstrates that by examining behavioral experiments with more details, it is possible to find minor alterations in behavior that can lead to significant discoveries, which could lead to earlier diagnoses in humans.

The pro-inflammatory cytokines that are released in response to CNS injury are involved in a number of neurodegenerative disorders. However, still little is known regarding the involvement of these factors in normal brain functioning. The study of Chai et al. examined the developmental profile in expression of the pro-inflammatory cytokine macrophage inhibitory factor (MIF) in hippocampus. Interestingly, in addition to glial precursor cells in dentate gyrus, MIF was expressed in parvalbumin- and reelin-expressing interneurons and involved in neurite outgrowth during hippocampal development.

Cinalli et al. examines the different roles that hippocampal CA1 and the Perirhinal cortex (Prh) play in spatial object memory. The authors had mice perform a novel object behavior task where they explored objects for different lengths of time to form “weak” or “strong” memories. For weak memories, the animals explored the objects for 10 s, for strong memories the animals explored the objects for 30 s. The animals were then tested 24 h later by replacing one of the objects. For one group of mice, the researchers bilaterally infused muscimol into either the CA1 or the Prh immediately after the learning session. During the test session, inactivation of CA1 impaired the mice from strong object memory, but not weak object memory. Inactivation of the Prh impaired weak object memory but not strong object memory. The researchers also examined the levels of the protein ARC, a protein that is involved with synaptic plasticity by using immunohistochemistry to examine protein and qPCR to examine the levels of mRNA. The researchers found that there were higher levels of ARC protein in CA1 after strong object memory compared to the Prh and higher levels of ARC protein in the Prh after weak object memory compared to CA1. ARC mRNA was significantly lower in the Prh during the strong object memory task, which may indicate that synaptic plasticity is reduced in the Prh during strong object memory formation. Overall, this paper provides evidence that CA1 is involved in the formation of strong object memories and Prh is involved in the formation of weak object memories. The authors suggest that these two processes are complementary and the two regions may work together in object memory formation.

Smith et al. demonstrates that unilateral and bilateral damage to the vestibular system causes spatial memory impairment and hippocampal place cell dysfunction, few studies have investigated the vestibular system's role in modulating hippocampal LTP and NMDA receptor expression. This review compares and evaluates *in vivo* and *in vitro* rodent studies investigating the nature of LTP in the hippocampus following vestibular lesions. The review cites evidence of decreased neuronal excitability *in vitro* following a unilateral vestibular lesion (UVL), but states *in vivo* studies found no such difference following a bilateral vestibular lesion (BVL). *In vivo* BVL studies were also inconsistent amongst each other, with contradictory findings in the hippocampal dentate gyrus subregion following LTP. Finally, one study found NMDA receptor subunit expression decreased following UVL, whereas another study found no change in NMDA receptor subunit expression after UVL. The review concludes that, using the body of literature available, it is difficult to form a cohesive view of the vestibular system's role in modulating hippocampal LTP due to inconsistent findings and multiple methodological differences between studies.

Although it is known that hippocampal oriens-lacunosum/moleculare (OLM) cells express hyperpolarization-activated cation channels (h-channels), it is unclear whether these h-channels are localized in OLM dendrites. Sekulić et al. constructed three computational multi-compartment models of OLM cells from the hippocampal CA1 region based on biophysical, morphological and h-channel parameters derived from experimental OLM cell recordings. After determining their models could correctly predict OLM cell biophysical properties, the authors combined electrophysiology together with a computational multi-compartmental model of OLM cells to identify h-channels in OLM cells based on their biophysical properties. Results demonstrated that their multi-compartment models of OLM cells needed h-channels present in the dendrites in order to be compatible with experimental data, suggesting h-channels are present in the dendrites of CA1 OLM cells. Overall, this work establishes that it is possible to combine experimental methods with computational modeling to characterize the biophysical properties of neurons.

Experiments performed by Memon et al. explore for the first time the possibility that in protein PTEN-induced kinase 1 (PINK1) KO rats, a model for autosomal recessive familial Parkinson's disease (PD), non-motor symptoms such as cognitive dysfunction might be affected by the lack of this protein. They tested this hypothesis evaluating short- and long-term plasticity along with the synaptic transmission in hippocampal CA3-CA1 pathway. Their results indicate that hippocampal plasticity is not affected by PINK1 loss, at an early age when motor symptoms are starting to appear and the excitatory transmission is already compromised in the striatum.

Glycine represents an important inhibitory influence in the brainstem and spinal cord *via* glycine-activated chloride channels, and GABA-A receptors medicate inhibition throughout the CNS. New modulators of these receptor classes could represent therapeutics. Bukanova et al. examined the effect of novel androstane and andrestene neuroactive steroids on both receptor types and found generally stronger and more potent effects on glycine receptors. Because glycine receptors are found on many brain cells, even those without glycinergic synapses, these compounds could be of widespread utility. Schaefer et al. investigated the role of glycine receptor mutations on anxiety and startle phenotypes. Kaouane et al. discuss the role of corticosterone and glucocorticoid receptor activation in the dorsal hippocampus with regard to post-traumatic stress disorder and fear memory. Meanwhile, Lu et al. re-examined the role of GABA-A receptor subclasses in mediating the impact of pregnane neurosteroids on inhibition. Using knock-in, pharmacoresistant mouse lines, they found, in contrast with some previous results, little evidence for subtype selectivity of allopregnanolone and allotetrahydrodeoxycorticosterone (THDOC). This could alter understanding of the impact of neurosteroids on behaviors, including mood.

Dawitz et al. investigated the role of GABA and glutamate in the emergence of spontaneous synchronized network activity (SSNA) and their role in the development of neuronal circuits during the 1st postnatal weeks. They confirm that in mouse medial entorhinal cortex during the 2nd postnatal week, SSNA persists and in fact peaks, and is dependent on ionotropic glutamatergic signaling. More specifically, the authors say: SSNA differs from that observed during the 1st postnatal week in two ways: First, the entorhinal cortex does not drive network activity in the hippocampus but only in the neighboring neocortex. Second, GABA does not drive network activity but influences it in a manner that is dependent both on age and receptor type. The authors conclude that while there is a partial mechanistic overlap in SSNA between the 1st and 2nd postnatal weeks, unique mechanistic features do emerge during the 2nd week, suggestive of different or additional functions of medial entorhinal cortex within the hippocampal-entorhinal circuitry with increasing maturation.

Altogether, this article collection addressed the latest hippocampal research and can be inspiring for the next Spring Hippocampal Research Conference in Verona (Italy) in 2023, which we are all looking forward to attending in person.

## Author Contributions

All authors listed have made a substantial, direct and intellectual contribution to the work, and approved it for publication.

## Conflict of Interest

The authors declare that the research was conducted in the absence of any commercial or financial relationships that could be construed as a potential conflict of interest.

## Publisher's Note

All claims expressed in this article are solely those of the authors and do not necessarily represent those of their affiliated organizations, or those of the publisher, the editors and the reviewers. Any product that may be evaluated in this article, or claim that may be made by its manufacturer, is not guaranteed or endorsed by the publisher.

